# Pulmonary Fungal Infections in a Tertiary Cancer Center: A Morphological Correlation of 160 Cases with CT and PET Imaging

**DOI:** 10.3390/diagnostics15172238

**Published:** 2025-09-03

**Authors:** Sebastian Lyos, Mylene T. Truong, Cesar A. Moran

**Affiliations:** M D Anderson Cancer Center, Houston, TX 77030, USA; slyos@yahoo.com (S.L.); mtruong@mdanderson.org (M.T.T.)

**Keywords:** lung, fungal, infectious, neoplasia, imaging

## Abstract

**Background:** Pulmonary fungal infections can mimic malignancies, especially in patients with a prior cancer diagnosis. This study presents 160 patients who were suspected to have malignancies but were diagnosed with fungal infections. **Methods:** Clinical, radiological, and histopathological features were recorded for all 160 patients. The patients included 61 women and 99 men, aged between 23 and 78 years (median age: 61 years). Diagnostic imaging identified either single or multiple pulmonary nodules. Tissue diagnosis was obtained in all cases, identifying various etiological agents, with *Histoplasma*, *Cryptococcus*, and *Aspergillus* being the top three infections. **Results:** Out of the 160 patients, 61 (38.1%) had a prior history of malignancy, and 29 (18.1%) had ongoing evidence of malignancy. Ninety-nine patients had no history of prior malignancy but presented with abnormal diagnostic imaging findings. The presence of single or multiple lesions in the lung, especially in patients with a history of malignancy, posed a diagnostic challenge, often raising the possibility of metastatic disease or primary lung malignancy. **Conclusions:** Patients with single or multiple pulmonary nodules, particularly those with a history of malignancy, should undergo tissue diagnosis to accurately define the process. This comprehensive assessment is crucial to determine whether the nodules are due to an infectious process or malignancy.

## 1. Introduction

The presence of single or multiple pulmonary nodules frequently poses diagnostic challenges, especially in patients with a history of malignancy. Similarities in the radiologic presentation of granulomatous infections and malignancies often lead to diagnostic challenges [1,2]. Although imaging modalities such as computed tomography (CT) and positron emission tomography (PET) provide excellent radiologic information, they are not specific for an unequivocal separation between infection and malignancy [3,4].

Fungal pathogens like *Histoplasma*, *Aspergillus*, and *Cryptococcus* are particularly notorious for mimicking neoplastic processes [5,6]. *Histoplasma capsulatum*, in endemic regions, can produce pulmonary nodules that persist and calcify, making radiologic differentiation from malignancy difficult [7]. Similarly, *Cryptococcus* and *Aspergillus* can form solitary or multiple pulmonary nodules with increased fluorodeoxyglucose (FDG) uptake on PET, raising false suspicion of malignancy [8,9]. Some authors have highlighted that invasive fungal infections can be misinterpreted as tumor progression in cancer patients [10]. While case reports underscore these diagnostic pitfalls [11,12], a retrospective analysis from the perspective of a cancer center is lacking. Our study analyzed a retrospective cohort of biopsy-confirmed fungal infections initially suspected to be malignant in which imaging was not specific, and tissue biopsies confirmed the diagnosis of fungal infection. In addition, it is important to highlight that often biopsy material is obtained without obtaining tissue cultures to correlate with microbiology or without having the benefit of prior antigen testing in urine or serum. Such challenges are frequently posed in the evaluation of pulmonary nodules or masses in patients with a prior history of malignancy and in which pulmonary biopsies do not unequivocally exclude malignancy or infection. We are fully aware that, by radiological means, the distinction of malignancy and fungal infection may not be possible, and we are similarly aware that the pathologist’s role is the morphological description of fungal elements. However, we are also aware that in daily practice, without the benefit of antigen testing or tissue cultures, pathologists offer an interpretation of the possible genus of the fungi by evaluating the morphology of fungal elements. Herein, we describe 160 cases in which such an approach was undertaken.

## 2. Materials and Methods

### 2.1. Study Design and Data Collection

This retrospective study reviewed cases from January 2018 to January 2024 involving patients who underwent biopsy for pulmonary nodules or masses following imaging suspicious of malignancy at a tertiary cancer center. This study was conducted following the guidelines from an approved IRB protocol. Inclusion criteria required histopathologic confirmation of fungal elements in the tissue evaluated and available radiologic interpretation. Extracted data included imaging impression, imaging modality, fungal species, final malignancy diagnosis, lesion size, and number.

### 2.2. Variable Classification

Different fungal infections were divided in cases in which there was the presence of yeast organisms of different sizes and hyphae. Morphologically, these organisms included *Histoplasma* spp., *Cryptococcus* spp., *Blastomyces* spp., *Coccidioides* spp., *Aspergillus* spp., and *Mucor* spp. Imaging impressions were grouped as malignancy, infection, or both. Imaging modalities were consolidated into PET or CT with or without intravenous contrast. Lesions were classified as single vs. multiple and measured for size.

### 2.3. Histological Analysis

In all cases, hematoxylin–eosin-stained sections were available for review, as were histochemical stains including Gomori Methenamine Silver (GMS), acid-fast bacilli (AFB), and mucicarmine.

### 2.4. Statistical Analysis

Descriptive statistics summarized frequencies and proportions. The false-positive rate was defined as the proportion of cases with an imaging impression of malignancy but no confirmed malignancy. Chi-square and Fisher’s exact tests assessed associations between imaging impressions and outcomes. Significance was set at *p* < 0.05. All analyses were conducted using R version 4.2.0.

## 3. Results

### 3.1. Clinical Features

The cohort included 160 patients, comprising 61 women and 99 men. The median age was 61 years (range: 23–86 years), and age distribution was similar across groups. Among those diagnosed with malignancy, the median age was also 61, with a slightly narrower age range of 23–78 years. While more males than females were found to have malignancy (21 vs. 8), statistical testing revealed no significant association between sex and malignancy status (χ^2^ = 1.17, *p* = 0.28). Similarly, there was no statistically significant difference in age between patients with and without malignancy (t = −0.36, *p* = 0.72). These findings suggest that demographic characteristics such as age and sex did not significantly influence the likelihood of a malignant diagnosis in this population.

In general, biopsy material was obtained from 127 patients, while 33 had surgical resection of the lung lesion. Among 160 patients with tissue-proven fungal infections, 29 (18.1%) were found to have ongoing evidence of malignancy at the time of diagnosis. The most common concurrent malignancies included lung adenocarcinoma (*n* = 14), head and neck squamous cell carcinoma (*n* = 3), pulmonary neuroendocrine neoplasms (*n* = 3), soft tissue sarcomas (*n* = 3), pulmonary small cell carcinoma (*n* = 2), colorectal carcinoma (*n* = 2), lymphoma (*n* = 1), and renal cell carcinoma (*n* = 1). When reviewing prior oncologic histories across the full cohort, the most frequent previous malignancies were also lung adenocarcinoma (*n* = 27), breast carcinoma (*n* = 12), lymphoma (*n* = 11), and renal cell carcinoma (*n* = 10), indicating a broad representation of solid and hematologic malignancies. Of the 29 patients with ongoing malignancy, 10 (34.5%) represented recurrence or the progression of previously diagnosed malignancy, while the remaining 19 (65.5%) were newly identified malignancies discovered concurrently with fungal infection.

Prior malignancy was noted in 61 patients (38.1%), with a particularly high rate among those with Aspergillus (55.9%) and Blastomyces (50%). These patients had received oncological treatment with either chemotherapy, radiation therapy, or both, depending on the original malignancy. These findings are presented in Table 1. This suggests possible immunosuppressive or treatment-related predispositions to fungal infections in oncologic patients. The relationship between prior malignancy and specific fungal pathogens may be influenced by geographic exposure, host immunity, and recent oncologic therapies.

More notable is the presence of a single pulmonary lesion in 86 patients, while multiple pulmonary lesions were identified in the remaining 74 patients, of which 8 had disease restricted to one lobe, 19 to one lung, and the remaining 47 had lesions in both lungs. In radiological terminology, a lung lesion less than 3 cm is a nodule (*n* = 110, of which 57 were round and 53 were oval). A lung lesion greater than 3 cm is a mass (*n* = 50, of which 33 were masses and 17 were mass-like consolidations). Nine patients had only consolidation, of which six were in the peripheral distribution in the subpleural location. Eight patients had both nodules and consolidation. Eleven patients had calcifications in the lung lesions, and the patterns of calcifications were as follows: four amorphous, three central coarse, two complete, one lamellated, and one speck. Thirty-three patients showed lesions with the hypodense sign, a central hypodensity in lung consolidation or nodule imaged by computed tomography, corresponding to a central area of necrosis [13]. Other signs included the halo sign (ground glass opacity surrounding a pulmonary nodule or mass, *n* = 9), reverse halo or atoll sign (ground glass opacity surrounded by a peripheral solid rim, *n* = 1), and cavity (*n* = 33) [14]. Of the 33 patients who had resection of lung lesion(s), 7 had lung cancer, 18 had metastatic disease, 3 had hematological malignancy (lymphoma, myeloma, or lymphoproliferative disorder), and 5 had infection only. Of 39 patients who had prior imaging studies for comparison, 32 patients had lesions that increased in size, while 7 patients had lesions that decreased in size. The presence of adenopathy (hilar only, *n* = 10; mediastinal only, *n* = 7; and mediastinal and hilar, *n* = 36), pleural effusion (unilateral, *n* = 6 and bilateral, *n* = 4), and pericardial effusions (*n* = 2) was observed, but it was not possible to determine if these findings were due to malignancy or infection. Of 70 patients who had F-18 fluorodeoxyglucose (FDG) PET/CTs, 51 had lung lesions that were FDG avid while 19 had lung lesions that were not FDG avid. Interpreting PET/CT imaging in patients with infection and inflammation can be challenging, as these conditions can be FDG avid and mimic malignant processes. The metabolic activity in immune cells, such as leukocytes, lymphocytes, and macrophages, can lead to false-positive findings that can confound PET/CT oncologic interpretation [15]. We could not identify any statistical significance between the size of the lesion and the histological result.

In surgical resections, tissue cultures were not obtained as the tissue was received in non-sterile conditions.

### 3.2. Histopathological Features

All cases were characterized by the presence of necrotizing granulomatous inflammation in different proportions. Also, the presence of entrapped vascular structures within the necrosis and the variable presence of inflammatory cells (lymphocytes, plasma cells, and eosinophils) were identified. The breakdown of these different infections and their association with malignancy is depicted Table 2. However, it is important to mention that, morphologically, the top three fungal organisms that were identified corresponded to *Histoplasma* (67 cases or 41.9%), *Cryptococcus* (40 cases or 25%), and *Aspergillus* (34 cases or 21.3%). However, out of the 160 patients evaluated, malignancy was confirmed in 26 patients (16%) who also had concurrent fungal infection.

The main histopathological feature in all cases was the presence of granulomatous inflammation. The use of GMS histochemical stains and mucicarmine were important in the final description of fungal elements, and morphologically those organisms were identified as follows.

**Figure 1:**
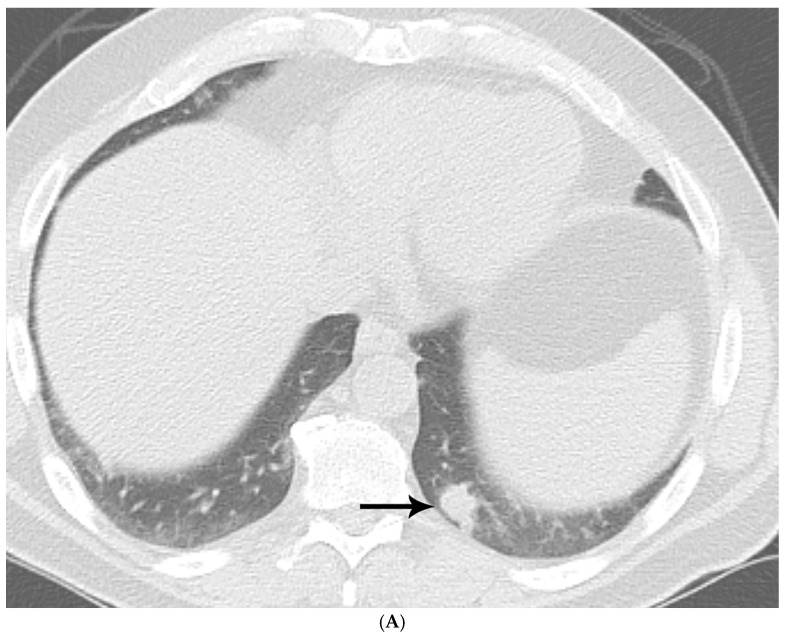

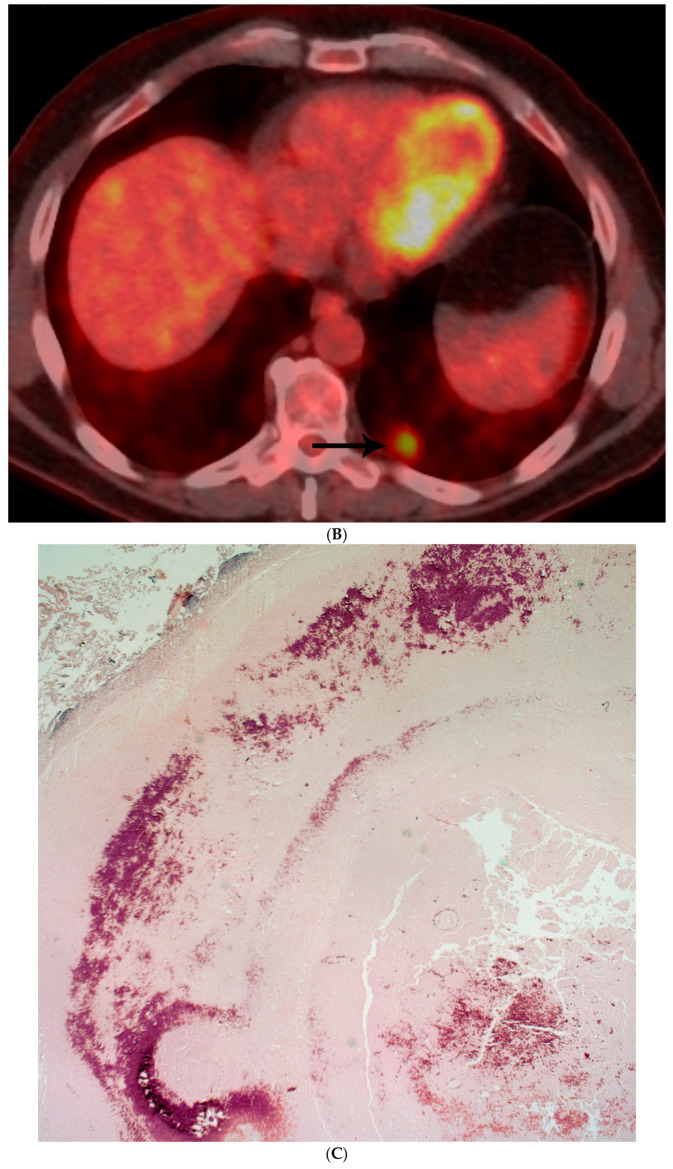

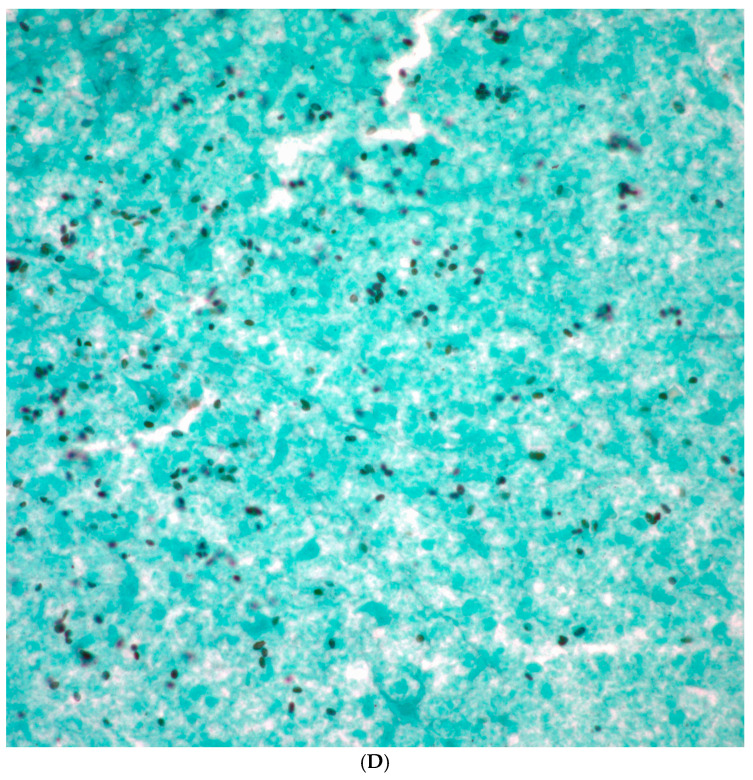
(**A**) CT shows a left lower lobe well-circumscribed 2 cm solid nodule (arrow); (**B**) PET/CT shows that the nodule (arrow) is FDG avid with a standard uptake value of five, suspicious for lung malignancy; (**C**) well-circumscribed necrotic nodule with focal areas of calcification; and (**D**) GMS stain showing numerous budding yeasts of *Histoplasma*.

*Histoplasma* spp.: Lesions appeared to have well-circumscribed borders, less cellularity, peripheral calcifications in some cases, and central necrosis. GMS histochemical stains showed the presence of numerous small budding yeasts organisms (Figure 1A–D).

**Figure 2:**
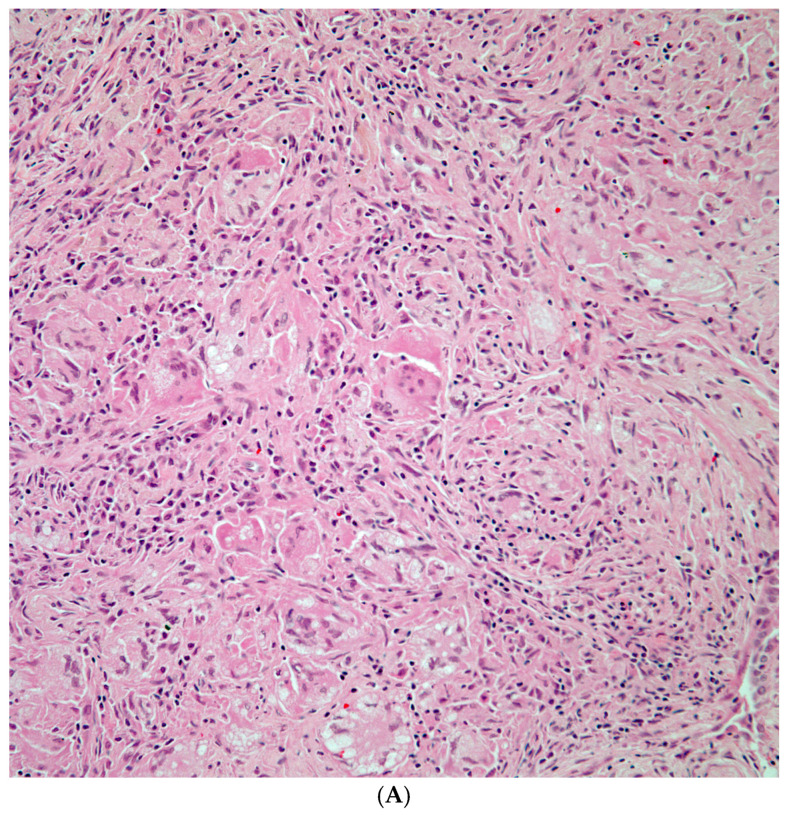

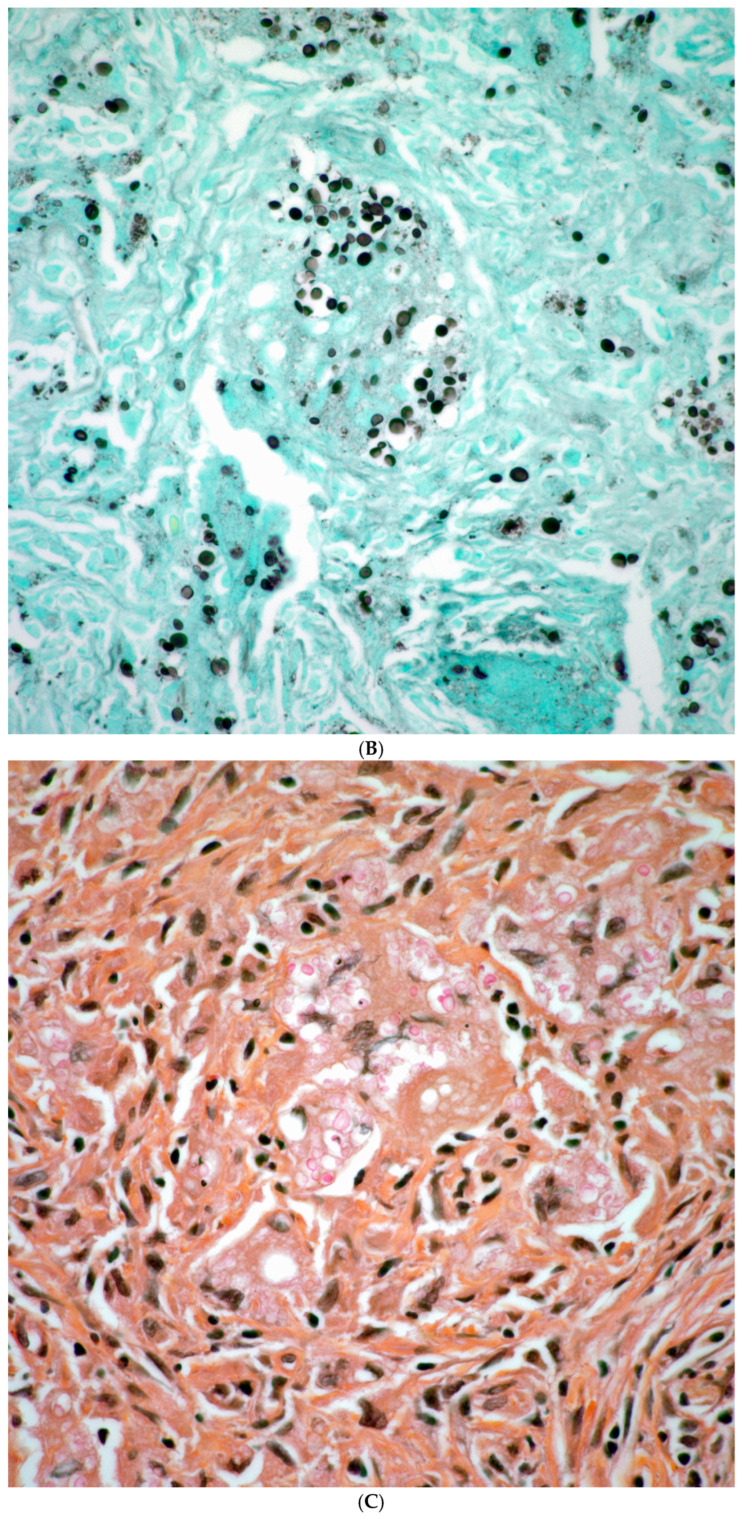
(**A**) Granulomatous inflammation replacing lung parenchyma with numerous giant cells; (**B**) GMS shows numerous fungal organisms; and (**C**) *Cryptococcus* spp. showing positive staining of the capsule with mucicarmine histochemical stain.

*Cryptococcus* spp.: Even though a granulomatous inflammation was present, lesions also showed more cellularity accompanied by inflammatory cells and multinucleated giant cells. Histochemical stains for GMS showed numerous organisms that also stained positively using mucicarmine stain (Figure 2A–C) and were morphologically compatible with *cryptococcus* spp.

**Figure 3:**
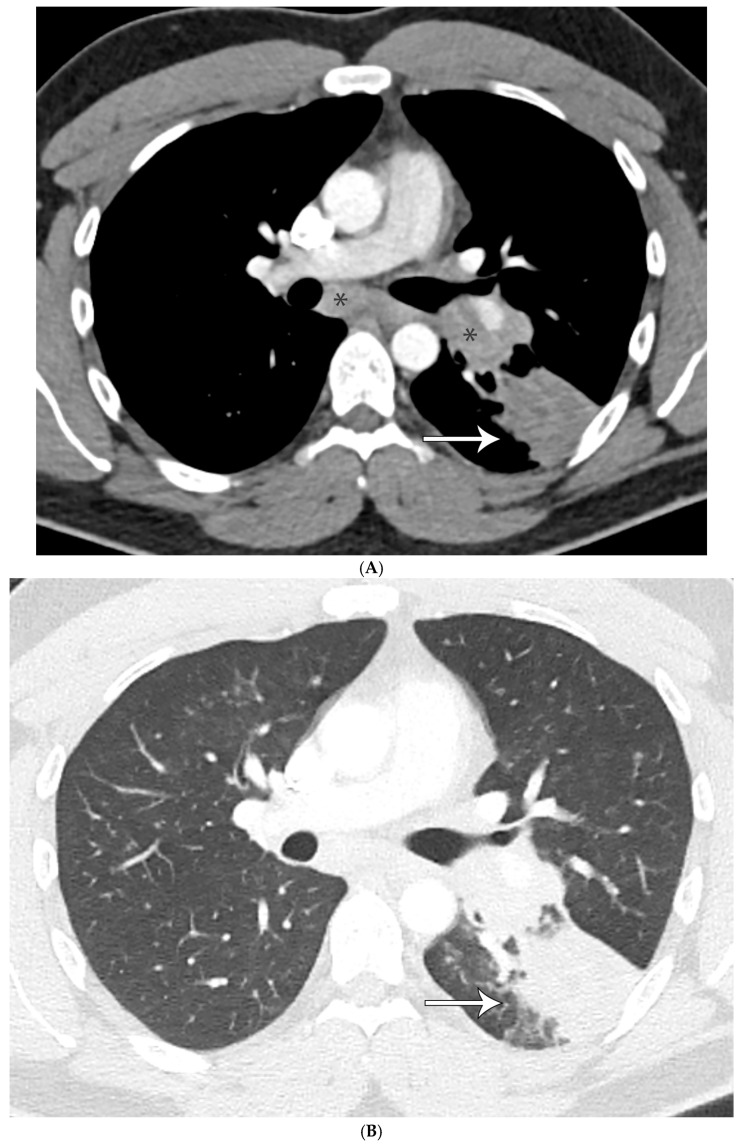

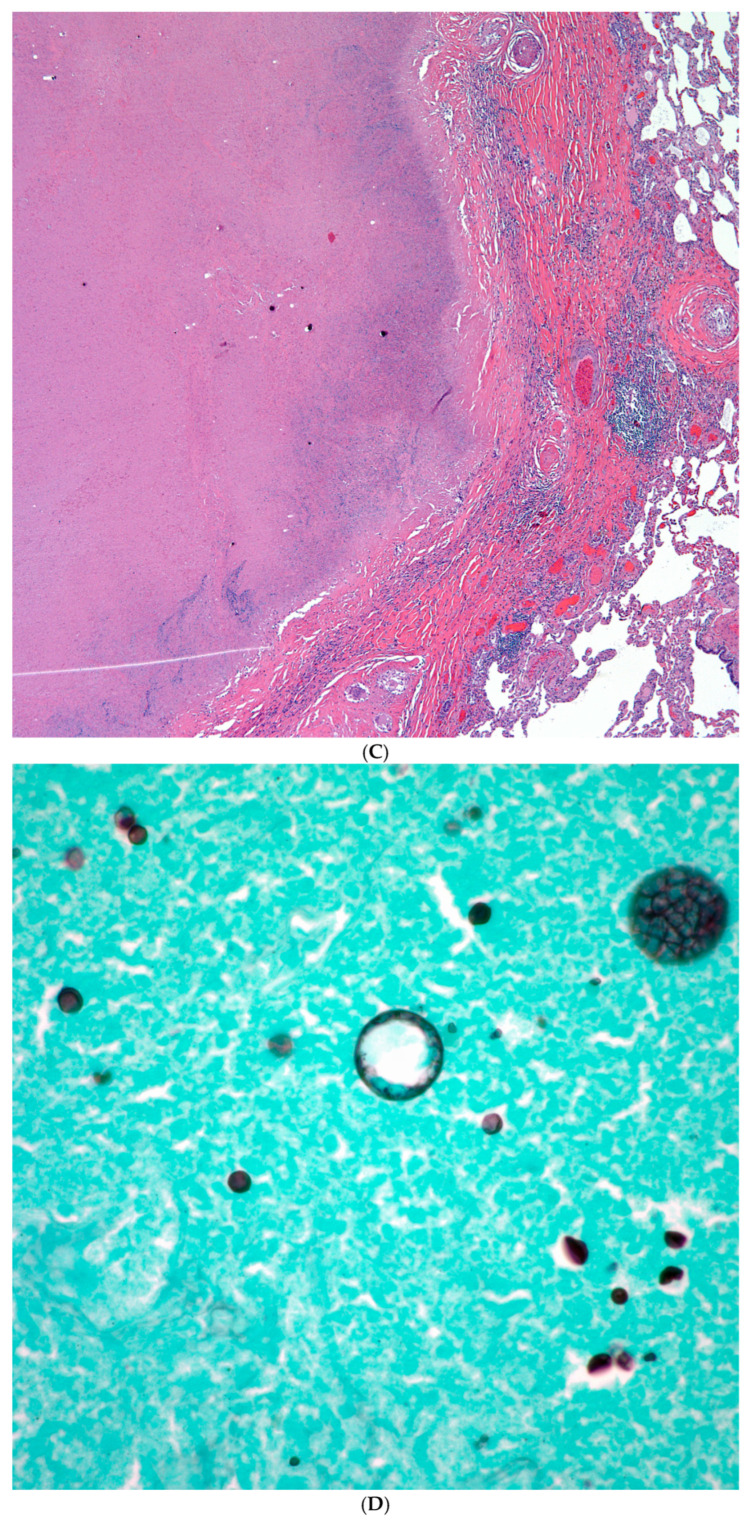
(**A**) CT shows left lower lobe 4 cm solid mass (arrow) abutting major fissure with adenopathy (asterisks) in left hilar and subcarinal regions mimicking lung malignancy. (**B**) Mass shows enhancement with administration of intravenous contrast and measures 80 Hounsfield units with lobular irregular contour on CT with lung windows. (**C**) Large necrotizing nodule replacing lung parenchyma. (**D**) GMS shows numerous large spherules containing endospores morphologically compatible with *Coccidioidomycosis* spp.

*Coccidioidomycosis* spp.: There were extensive areas of necrosis, and in conventional hematoxylin–eosin sections there were some fainted larger structures in some areas that on GMS stain showed the presence of large spherules with endospores morphologically in keeping with coccidioidomycosis (Figure 3A–D).

**Figure 4:**
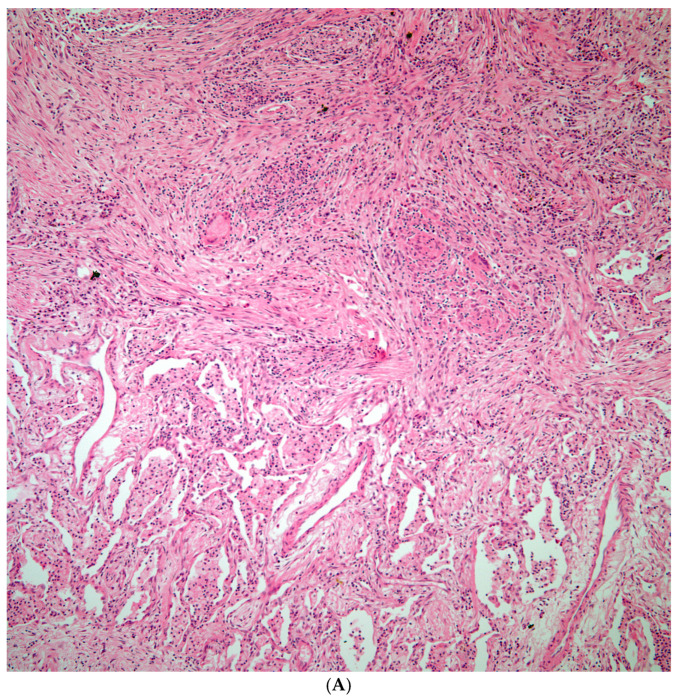

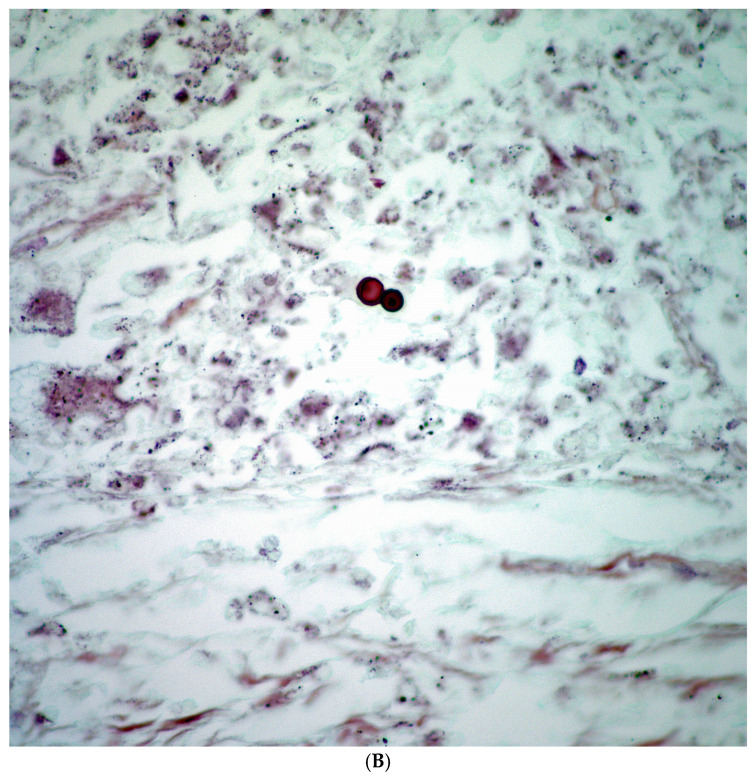
(**A**) Necrotizing granulomatous inflammation destroying lung parenchyma. (**B**) GMS shows scattered broad-based budding organisms morphologically compatible with *Blastomycosis* spp.

*Blastomycosis* spp.: The lung parenchyma was replaced by extensive areas of necrosis. Histochemical stains for GMS show the presence of broad budding yeast morphologically compatible with *Blastomyces* spp. (Figure 4A,B).

*Aspergillus* spp. was characterized by the presence of extensive areas of necrosis, which in focal areas showed the presence of slender septated hyphae, which on GMS histochemical stains became more prominent.

We also identified one case characterized by extensive areas of necrosis, and by GMS histochemical stain showed the presence of scattered broad septated hyphae, morphologically compatible with *Mucor* spp.

**Figure 5:**
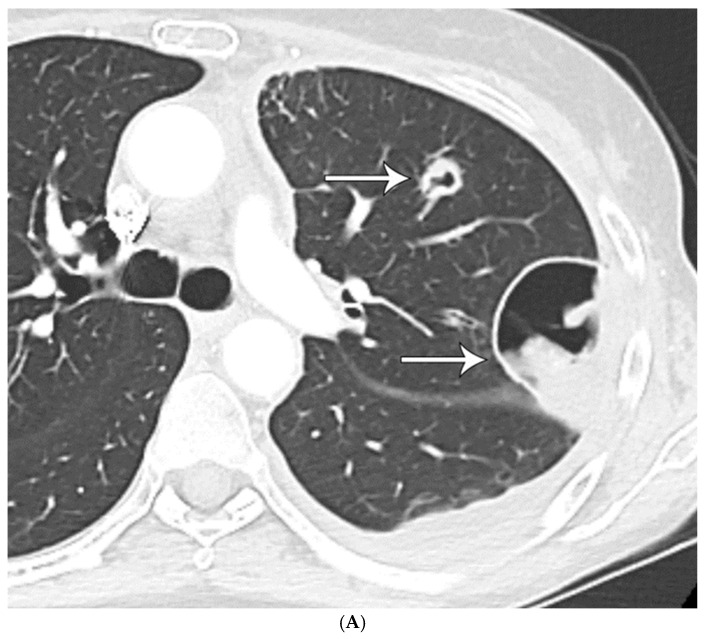

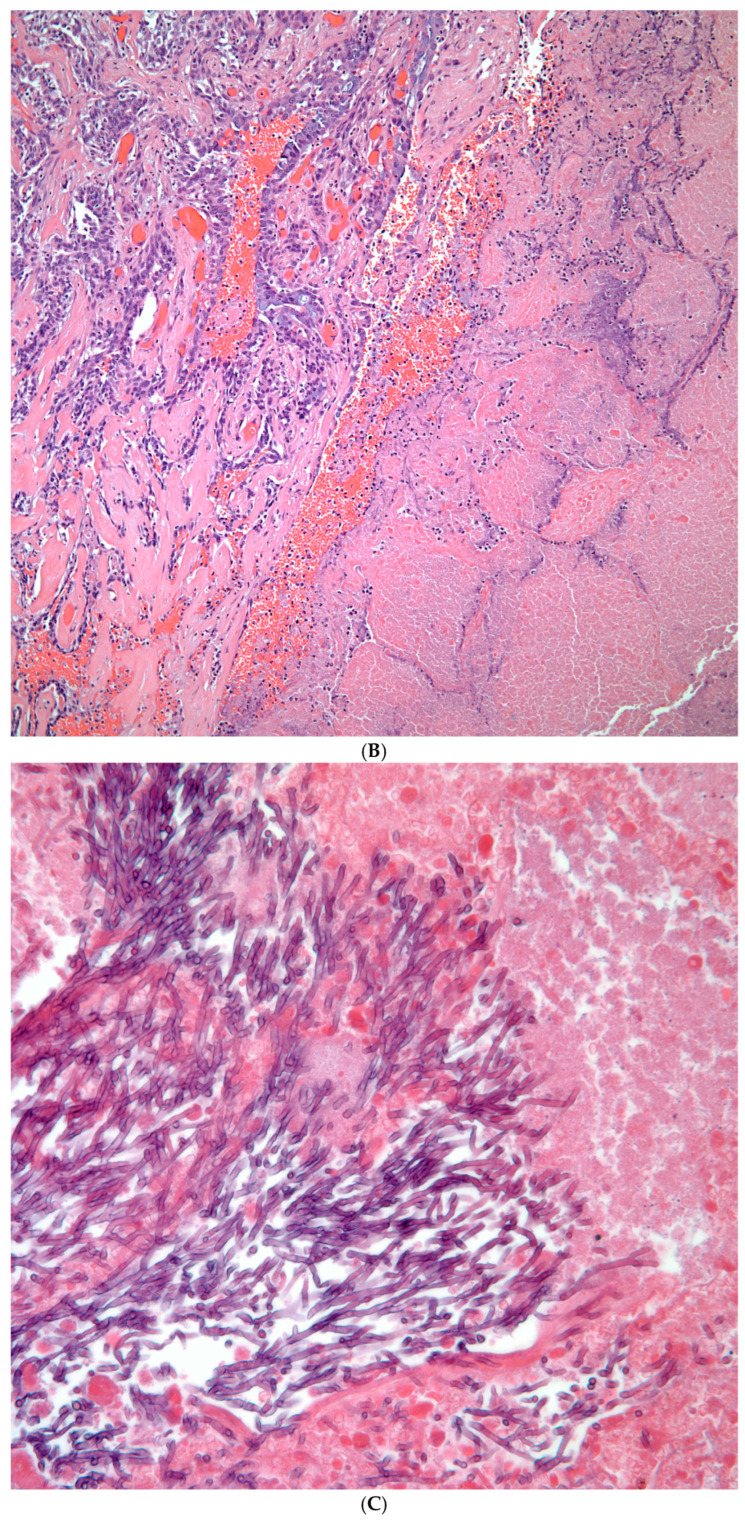
(**A**) Multiple cavitary lung lesions (arrows) in the left upper lobe of the lung. (**B**) Large necrotizing lesion replacing lung parenchyma showing dual association of carcinoma. (**C**) Branching slender septated hyphae, morphologically compatible with *Aspergillus* spp.

On the other hand, in 24 patients we were able to observe the presence of a neoplastic process concurrent with a fungal infection (Figure 5A–C).

## 4. Discussion

The unequivocal separation of infectious processes and malignancy in pulmonary lesions represents a challenge for which obtaining surgical tissue sampling for diagnosis is most often required. It is understood that in a more general clinical setting, for cases in which an infectious process is the top differential diagnosis, serum and urine antigen testing and tissue cultures may be procured to define the process. However, for patients who are being followed for any type of malignancy or in whom an infectious process is not suspected, these clinical tests may not be available at the onset. Therefore, there is a need for tissue diagnosis. Previous authors have also highlighted such diagnostic challenges and particularly highlighted Histoplasma as one of the leading fungal infections that may mimic malignancy [5,7,8]. Even though there have been advances in diagnostic imaging with the use of PET/CT to evaluate indeterminate pulmonary nodules, this imaging modality had the highest FPR at 86%, reaffirming concerns about their specificity in endemic and non-endemic fungal regions [3]. False positives are frequently driven by fluorodeoxyglucose (FDG) uptake in granulomatous infections, as observed in histoplasmosis, blastomycosis, and cryptococcosis. In one study, fungal infections accounted for 66% of false positives on integrated PET/CT scans [16]. The limited specificity of FDG-PET in endemic areas has also been quantified by Deppen et al. [17], who found significantly reduced diagnostic accuracy in such a context. CT scans with and without intravenous contrast showed similarly high FPRs, highlighting that even with contrast enhancement, fungal lesions often remain indistinguishable from malignancies [6,9]. Different studies have highlighted the challenge of separating fungal infection from neoplasia [18,19,20,21].

Our study also highlighted the radiological diagnostic challenge between pulmonary fungal infections and malignancies, which prompted the need to obtain tissue sampling to confirm diagnosis. In addition, it is important to highlight that the assessment of patients in a cancer center, where patients are mainly being followed for previous malignancies, creates a setting in which malignancy needs to be excluded. Our results reaffirm prior observations that solitary lung lesions raise higher suspicion for malignancy. Solitary lesions were not only more frequently suspected to be malignant but also had a higher false-positive rate (81.3%) compared to multiple lung lesions (68.2%). This is consistent with established diagnostic heuristics, where single, larger nodules are more likely to be interpreted as neoplastic [22,23]. However, lesion multiplicity and smaller size may point toward infectious etiologies, especially in immunocompetent patients with relevant exposures [23]. However, larger nodules are generally more concerning for malignancy, especially when accompanied by irregular borders or high FDG uptake [22,24]. Nevertheless, in endemic areas for granulomatous disease, infectious nodules can present with deceptively large sizes, sometimes exceeding 7 cm, and still be benign [17]. Even though our cohort was small, we observed a wide standard deviation and overlap in size distributions further highlighting that lesion size alone is an unreliable discriminator between fungal infection and malignancy. These findings reinforce the importance of a multimodal approach—combining imaging, clinical context, and histopathology—to reduce unnecessary invasive procedures prompted by size alone. It is also highly important to underscore the fact that at least 15% of the patients herein presented had both concomitant fungal infection and malignancy. This fact highlights the issue that in a cancer center or in patients being followed for any malignancy, pulmonary lesions, regardless of the size, need to be carefully evaluated and sampled to determine the possible etiology. However, in small biopsies the possibility of encountering both pathological processes may not be possible. In such cases, clinical and radiological suspicion may play a larger role in determining what conduct to follow with these patients and whether a different surgical procedure is warranted. Also important to highlight is that over the period of six years (2018–2024) that we were able to review, the existence of 160 patients with fungal infections is a small percentage in comparison to the thousands of cases of primary and metastatic disease that we see on a yearly basis. Thus, we consider that the number of fungal pulmonary nodules present in a cancer center setting is dramatically less than the number of malignancies seen, but that is relative to the population sampled.

In short, we have herein presented 160 patients with and without a prior history of malignancy and for whom diagnostic imaging was suspicious for malignancy. Even though all these patients had PET and CT modalities, which prompted obtaining surgical material for diagnosis, in only 26 patients (16%) was the diagnosis of dual infectious process and malignancy observed, while in 136 patients (85%) only infectious processes were documented. Nevertheless, this study also highlights that even in cases in which either a malignancy or infectious process alone is documented, the possibility of a dual process cannot be completely excluded. In these cases, we consider that strong clinical and radiological suspicion play a larger role in defining the conduct to follow and determining a possible surgical intervention. The perspective of the assessment of patients in a cancer center also needs to be considered, as the conduct in obtaining surgical material for diagnosis becomes a priority. As we stated earlier, in cases in which the top differential diagnosis is an infectious process, antigen testing in serum or urine and/or tissue cultures should be sought. However, once the material has become a surgical resection, it is the role of the pathologist to describe the morphology of the fungal elements and based on that morphology to attempt to provide the genus of the fungi, knowing of course that tissue cultures represent the gold standard to provide a final interpretation regarding the specific genus.

## 5. Conclusions

- By imaging, the distinction between an infectious process and neoplasia remains a diagnostic challenge.
- Even though tissue cultures and antigen testing in plasma and/or urine are important, often these are not necessarily available, but should be part of the overall assessment of patients with pulmonary nodules.
- Careful assessment of surgical material becomes highly important in identifying a fungal organism.
- Special histochemical stains (GMS) are recommended, and when needed, PCR studies are recommended.
- Morphological description of the fungal organism is an important role of the pathologist.
- In a small proportion of cases, the infectious process may also be associated with a neoplastic process.
- Clinicians should maintain a low threshold for fungal testing in patients with pulmonary nodules.

## Figures and Tables

**Table 1:** Sixty-one patients with prior history of malignancy and associated fungal infection.

Fungal Species	Patients with Prior Malignancy (*n*)	% of Species Cohort
*Histoplasma* spp.	21	31.3%
*Cryptococcus* spp.	15	37.5%
*Aspergillus* spp.	19	55.9%
*Coccidioides* spp.	4	28.6%
*Blastomyces* spp.	2	50.0%
*Mucor* spp.	0	0%
Total	61	38.1% of all cases

**Table 2:** Fungal species distribution and its association with malignancy.

**Fungal Species**	**Cases (*n*)**		**Percentage (%)**	
*Histoplasma* spp.	67		41.9%	
*Cryptococcus* spp.	40		25.0%	
*Aspergillus* spp.	34		21.3%	
*Coccidioides* spp.	14		8.8%	
*Blastomyces* spp.	4		2.5%	
*Mucor* spp.	1		0.6%	
**Fungal Species**	**Total Cases (*n*)**	**With Malignancy (*n*)**	**Fungal Only (*n*)**	**% with Malignancy**
*Histoplasma* spp.	67	5	62	7.5%
*Cryptococcus* spp.	40	7	33	17.5%
*Aspergillus* spp.	34	6	28	17.6%
*Coccidioides* spp.	14	4	10	28.6%
*Blastomyces* spp.	4	2	2	50.0%
*Mucor* spp.	1	0	1	0%
Total	160	24	136	15.0%

## Data Availability

The data available in the elaboration of this manuscript is presented already in the manuscript.
